# Structural connections support emotional connections: Uncinate Fasciculus microstructure is related to the ability to decode facial emotion expressions

**DOI:** 10.1016/j.neuropsychologia.2017.11.006

**Published:** 2020-08

**Authors:** Bethany M. Coad, Mark Postans, Carl J. Hodgetts, Nils Muhlert, Kim S. Graham, Andrew D. Lawrence

**Affiliations:** a Cardiff University Brain Research Imaging Centre (CUBRIC), School of Psychology, Cardiff University, UK; b Division of Neuroscience & Experimental Psychology, Faculty of Biology, Medicine and Health, University of Manchester, Manchester, UK

**Keywords:** Anterior temporal lobe, Diffusion MRI, Emotion, Facial expression, Orbital and medial prefrontal cortex, Uncinate Fasciculus

## Abstract

The Uncinate Fasciculus (UF) is an association fibre tract connecting regions in the frontal and anterior temporal lobes. UF disruption is seen in several disorders associated with impaired social behaviour, but its functional role is unclear. Here we set out to test the hypothesis that the UF is important for facial expression processing, an ability fundamental to adaptive social behaviour. In two separate experiments in healthy adults, we used high-angular resolution diffusion-weighted imaging (HARDI) and constrained spherical deconvolution (CSD) tractography to virtually dissect the UF, plus a control tract (the corticospinal tract (CST)), and quantify, via fractional anisotropy (FA), individual differences in tract microstructure. In Experiment 1, participants completed the Reading the Mind in the Eyes Task (RMET), a well-validated assay of facial expression decoding. In Experiment 2, a different set of participants completed the RMET, plus an odd-emotion-out task of facial emotion discrimination. In both experiments, participants also completed a control odd-identity-out facial identity discrimination task. In Experiment 1, FA of the right-, but not the left-hemisphere, UF was significantly correlated with performance on the RMET task, specifically for emotional, but not neutral expressions. UF FA was not significantly correlated with facial identity discrimination performance. In Experiment 2, FA of the right-, but not left-hemisphere, UF was again significantly correlated with performance on emotional items from the RMET, together with performance on the facial emotion discrimination task. Again, no significant association was found between UF FA and facial identity discrimination performance. Our findings highlight the contribution of right-hemisphere UF microstructure to inter-individual variability in the ability to decode facial emotion expressions, and may explain why disruption of this pathway affects social behaviour.

## Highlights

• We studied white matter microstructure correlates of facial emotion decoding skills.
• Focused on the role of a key limbic tract, the Uncinate Fasciculus (UF).
• Right UF microstructure linked to facial expression decoding skills.
• UF microstructure not related to facial identity discrimination skills.
• Right UF has a distinct role in the processing of facial expressions of emotion.

## 1 Introduction

The Uncinate Fasciculus (UF) is a hook-shaped cortico-cortical white matter pathway that provides bidirectional connectivity between the orbital and medial prefrontal cortex (OMPFC), and the anterior portions of the temporal lobe (ATL), including the temporal pole, perirhinal cortex and amygdala (Petrides and Pandya, 2007, Schmahmann et al., 2007, Thiebaut de Schotten et al., 2012). Disruption of the UF is seen in a range of neurological and psychiatric conditions that are characterised by altered social behaviour, including autism spectrum disorder (ASD) (Kumar et al., 2010, Pugliese et al., 2009), frontotemporal dementia (FTD) (Mahoney et al., 2014, Whitwell et al., 2010), psychopathy (Craig et al., 2009, Sundram et al., 2012), and social anxiety disorder (Baur et al., 2013, Phan et al., 2009). By virtue of its connectivity, the UF has been suggested to underpin a ‘temporo-amygdala-orbitofrontal network’ (Catani et al., 2013) or ‘anterior temporal system’ (Ranganath and Ritchey, 2012), potentially critical to the regulation of social and emotional behaviour (Von Der Heide et al., 2013), but the precise functions of this network are unspecified. Here, we test the specific hypothesis that a critical role for the UF is in the decoding of facial expressions of emotion.

In humans, the face is the primary canvas used to express emotions nonverbally (Ekman, 1965). Given that human social interactions are replete with emotional exchanges, facial emotion processing abilities are crucial for the regulation of interpersonal relationships and for social functioning more generally (Fischer and Manstead, 2008). Facial emotion processing is a critical mechanism by which observers respond to others empathically, and also modify their own behaviour adaptively based on others’ facial signals (Matsumoto et al., 2008). Indeed, individual differences in the ability to decode facial expressions are linked to several indicators of social success (Elfenbein et al., 2007, Momm et al., 2015, Woolley et al., 2010).

Work in nonhuman primates originally identified the anterior temporal and prefrontal cortical areas as being important in the control and regulation of social behaviour (Franzen and Myers, 1973, Kling and Steklis, 1976; see Machado and Bachevalier, 2006 for later refinements). Subsequent neuropsychological investigations in humans found that damage to both ATL (including amygdala) (Adolphs et al., 2003, Adolphs et al., 2001b, Adolphs et al., 1999, Adolphs et al., 1996, Anderson et al., 2000, Cancelliere and Kertesz, 1990, Schmolck and Squire, 2001) and OMPFC (Dal Monte et al., 2013, Heberlein et al., 2008, Hornak et al., 1996, Tsuchida and Fellows, 2012, Shamay-Tsoory et al., 2009) was associated with impaired facial emotion decoding, particularly when right hemisphere (RH) ATL and OMPFC were damaged (Adolphs, 2002). Further, this was particularly evident for negative, relative to positive, valence emotions (Adolphs et al., 2001a, Damasio and Adolphs, 2003). Given the dynamic and complex nature of social interaction, rapid decoding of facial expressions requires the efficient transfer of information between these distributed temporal and frontal cortical regions (Adolphs, 2002, Vuilleumier and Pourtois, 2007). White matter tracts are the brain structures that allow such efficient, synchronised transfer of information across distant brain regions (Fields, 2008, Mesulam, 2012). As such, the ability of frontotemporal brain regions to communicate with one another via the UF may be critical for performance in tasks that challenge this critical domain of social-emotional functioning.

There is preliminary neuropsychological evidence that lesions impacting, but not selective to, the UF can lead to impaired emotion recognition (Fujie et al., 2008, Ghosh et al., 2012, Mike et al., 2013, Oishi et al., 2015). The role of the UF in facial emotion processing, however has yet to be systematically investigated. Here, across two separate experiments, we utilized an individual differences approach (Yovel et al., 2014) to isolate the functional contribution of the UF to the decoding of facial emotion expression in healthy adults.

In Experiment 1, participants completed the Reading the Mind in the Eyes Test (RMET) (Baron-Cohen et al., 2001). While initially developed for use in ASD, the RMET has been shown to be a valid and sensitive measure of subtle individual differences in facial emotion processing in healthy individuals (Baron-Cohen et al., 2001, Vellante et al., 2013). The RMET requires participants to select a verbal label that best describes the mental state being expressed in a series of images of the eye region of the face. Previous work has shown RMET performance to be sensitive to focal lesions of both the ATL and the OMPFC (Adolphs et al., 2002; Shaw et al., 2005). Furthermore, deficits in performance on the RMET have been reported in conditions associated with UF abnormalities, including ASD (Baron-Cohen et al., 2001, Losh and Piven, 2007); psychopathy (Ali and Chamorro-Premuzic, 2010, Sharp and Vanwoerden, 2014); and FTD (Baez et al., 2014, Gregory et al., 2002). Participants in Experiment 1 also completed an ‘odd-identity-out’ test of facial identity discrimination to control for non-emotional facial perceptual ability (Hodgetts et al., 2015). In Experiment 2, a separate group of participants completed the RMET and the odd-identity-out task, as well as an ‘odd-expression-out’ test of facial emotion discrimination (Palermo et al., 2013), analogous to the odd-identity-out task, which eliminated the linguistic requirements of the RMET.

To isolate the role of UF white matter in these tasks, we used high angular resolution diffusion-weighted imaging (HARDI) and constrained spherical deconvolution (CSD) tractography, which permits tracking through regions of crossing fibres (Tournier et al., 2008). Using this approach, we were able to virtually dissect (Catani et al., 2002) the UF in healthy adult participants and quantify, via fractional anisotropy (FA) (Basser, 1997), inter-individual variation in its microstructure. UF FA values were then correlated with inter-individual differences on our tasks of facial emotion and identity processing, to assess the behavioural relevance of individual differences in UF microstructure (Kanai and Rees, 2011). Increases in FA are typically associated with microstructural properties that support the efficient transfer of information along white matter tracts (Beaulieu, 2002). We therefore hypothesized that higher FA values in the UF, especially in the RH (Adolphs, 2002, Damasio and Adolphs, 2003), but not FA of a control tract (the corticospinal tract, CST), would be associated with better facial emotion decoding ability, but not facial identity discrimination ability. The results of the two studies supported this hypothesis.

## 2 Material and methods

### 2.1 Participants

Participants were scanned using an identical diffusion-weighted magnetic resonance imaging (dMRI), sequence in both experiments. Across the two experiments, data were collected from a total of 86 participants, all of whom self-reported as being healthy and having no history of psychiatric or neurological illness. Experiment 1 comprised 42 individuals (aged 19–40 years, Mean ± SD = 24 ± 6, 9 males) and Experiment 2 comprised a separate set of 44 individuals (aged 18–34 years, Mean ± SD = 24 ± 4; 14 males). All participants provided written informed consent prior to participation. The study was conducted in line with the Declaration of Helsinki and was approved by the Cardiff University School of Psychology Research Ethics Committee.

### 2.2 MRI data acquisition

Imaging data were collected at the Cardiff University Brain Research Imaging Centre (CUBRIC) using a 3 T GE HDx MRI system (General Electric Healthcare, Milwaukee, WI) with an 8-channel receive-only head RF coil. Whole brain HARDI data (Tuch et al., 2002) were acquired using a diffusion-weighted single-shot echo-planar imaging (EPI) pulse sequence with the following parameters: TE = 87 ms; voxel dimensions = 2.4 × 2.4 × 2.4 mm^3^; field of view=23 × 23 cm^2^; 96 × 96 acquisition matrix; 60 contiguous slices acquired along an oblique-axial plane with a slice thickness of 2.4 mm and no between-slice gap. To reduce artefacts arising from pulsatile motion, acquisitions were cardiac-gated using a peripheral pulse-oximeter. Gradients were applied along 30 isotropically distributed directions with b = 1200 s/mm^2^. Three non-diffusion-weighted images (DWI) with b=0 s/mm^2^ were also acquired according to an optimized gradient vector scheme (Jones et al., 1999). In addition, high-resolution anatomical images were acquired using a standard T1-weighted 3D FSPGR sequence comprising 178 axial slices (TR/TE = 7.8/3.0 s, FOV = 256 × 256 × 176 mm, 256 × 256 × 176 data matrix, 20 ° flip angle, and 1 mm isotropic resolution).

### 2.3 Diffusion MRI pre-processing

*ExploreDTI*_4.8.3 (Leemans et al., 2009) was used to correct for subject head motion and eddy current distortions. To correct for partial volume artefacts arising from voxel-wise free water contamination, the two-compartment ‘free water elimination’ procedure was implemented (Pasternak et al., 2009) yielding voxelwise maps of free water-corrected fractional anisotropy (FA). FA reflects the extent to which diffusion is anisotropic, or constrained along a single axis, and can range from 0 (fully isotropic) to 1 (fully anisotropic) (Basser, 1997).

#### 2.3.1 Tractography

Tractography (Conturo et al., 1999) was performed in native diffusion space in *ExploreDTI*, using deterministic tracking based on CSD (Tournier et al., 2004), which extracts peaks in the fibre orientation density function (fODF) in each voxel. The fODF quantifies the proportion of fibres in a voxel pointing in each direction and so information about more complex fibre configurations can be extracted (Jones et al., 2013). This approach was chosen as the most appropriate technique for reconstruction of the UF, because of its proximity to other WM tracts (e.g. the anterior commissure) leading to crossing/kissing fibre combinations (Ebeling and von Cramon, 1992). For each voxel, streamlines were initiated along any peak in the fODF that exceeded an amplitude of 0.1 (hence, multiple fibre pathways could be generated from any voxel). Each streamline continued, in 0.5 mm steps, following the peak in the ODF that subtended the smallest angle to the incoming trajectory (subject to a threshold of 60° to prevent the reconstruction of anatomically implausible fibres). Once whole-brain tractography was complete, regions-of-interest (ROIs) were used to virtually dissect the UF and the CST. The resulting tract masks were intersected with the voxelwise free-water corrected whole-brain FA map to obtain tract-specific free-water corrected measures of FA.

#### 2.3.2 Uncinate Fasciculus (UF)

**Fig. 1:**
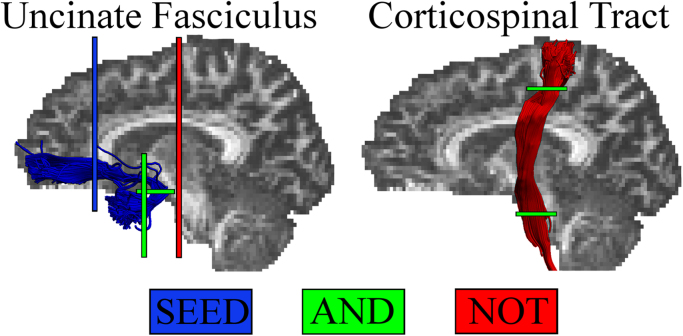
Example reconstruction of the Uncinate Fasciculus (UF) and corticospinal tract (CST) from a single participant. The waypoint regions-of-interest (ROIs) used for reconstructing each tract are shown.

Three-dimensional reconstruction of the UF was performed using a multiple region of interest (ROI) approach, extracting the left and right UF separately. ROIs were manually drawn in native diffusion space on colour-coded fibre orientation maps (Pajevic and Pierpaoli, 1999), using landmark techniques previously shown to be highly reproducible (Catani and Thiebaut de Schotten, 2008, Wakana et al., 2007). The UF was delineated as described in Catani and Thiebaut de Schotten (2008). A SEED ROI was drawn on a coronal slice located just anterior to the corpus callosum in the inferior medial region where the UF enters the frontal lobe (see Fig. 1). Two AND gates were then placed in the temporal lobe, one gate encompassed the whole of the white matter of the ATL and was drawn on a coronal slice located just anterior to where the temporal lobe meets the frontal lobe. The other gate was drawn on an axial slice located in line with the upper portion of the pons, around a bundle of fibres oriented in the superior-inferior direction in the temporal white matter, capturing the region where the UF curves around the Sylvian fissure. Two NOT ROI gates were then placed to exclude fibres from other tracts. One NOT ROI was placed on a coronal slice located posterior to the pons, the gate covered the entire brain to prevent fibres from the inferior fronto-occipital fasciculus (IFOF) from being included. A second NOT gate was placed on a sagittal slice located between the two hemispheres. The gate covered the entire brain and was placed to ensure no fibres were included from commissural tracts such as the anterior commissure. Each tract was visually inspected to ensure the tract was consistent with the UF and did not include any erroneous fibres. Additional NOT gates were placed to remove any fibres which were inconsistent with the known path of the UF.

#### 2.3.3 Corticospinal Tract (CST)

To confirm the anatomical specificity of any effects in the UF, the above tractography protocol was additionally carried out to extract FA indices from both the left and right hemisphere of a major motor system tract, the CST (Lemon, 2008). The CST was extracted as described previously (Wakana et al., 2007). The CST was defined as the tract running between the primary motor cortex and the mid-brain (Farquharson et al., 2013, Thiebaut de Schotten et al., 2011). An AND gate was placed in an axial slice located just superior to the superior colliculus, this gate encompassed the entire cerebral peduncle on either the left or the right side depending on the tract being extracted (see Fig. 1). A second AND gate encompassed the anterior branch of the CST just superior to where the tract splits to run either side of the central sulcus. As with the protocol for the other tracts, NOT gates were placed to exclude fibres clearly belonging to tracts other than the CST.

### 2.4 Tasks and procedure

#### 2.4.1 Experiment 1

##### 2.4.1.1 Reading the Mind in the Eyes Test (RMET)

**Fig. 2:**
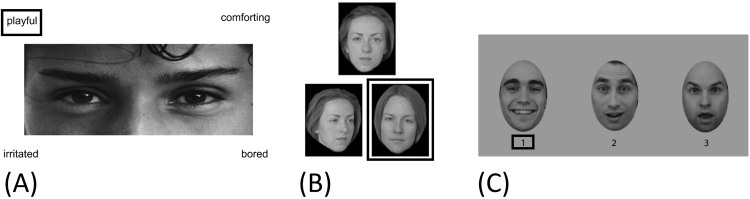
Example trials from each of the experimental tasks. (A) Reading the Mind in the Eyes Test; (B) Odd-Identity-Out Task; (C) Odd-Emotion-Out Task. For each example, the target stimulus is identified.

Participants completed the RMET (Baron-Cohen et al., 2001) as part of a larger testing battery. The RMET is a reliable and sensitive measure of subtle individual differences in face-based mental state decoding in the typical population, without being susceptible to floor or ceiling effects (Vellante et al., 2013). The RMET consists of 36 greyscale photographs, cropped to depict the eye region of a series of adult faces, each displaying a different complex mental state (e.g. *guilt, curiosity*). Images are surrounded at each corner by four mental state terms (1 target and 3 foils) and for each image participants are required to correctly select the word presented that best describes the mental state being expressed (see Fig. 2A for a sample item). A pen and paper version of this task was utilized and no time limit was imposed. Taken from magazine photos, images were standardized to a size of 11.5 cm by 4.5 cm; cropped such that each image displayed the eye region from just above the eyebrow to halfway down the bridge of the nose; and displayed on a white background (Baron-Cohen et al., 2001). As such, the RMET images have only limited standardization in terms of lighting, etc. but are highly naturalistic. A glossary with definitions of the mental state terms and examples of their use was provided to reduce potential confounding with vocabulary skills. Participants score 1 point for each correct answer, and test scores were calculated as the total number of correct responses (maximum score 36) and then converted to percentage correct values. The 36 eyes stimuli have previously been classified into three valence categories: positive (8 trials, e.g., *playful),* negative (12 trials, e.g., *upset*) and non-emotional/cognitive (16 trials, e.g., *insisting*) (Harkness et al., 2005). In addition to total scores, we therefore also computed total scores for cognitive and affective mental state items separately, again expressed as percentage correct values (see Sections 3.1.3.2 for further comment)

##### 2.4.1.2 Facial identity discrimination

A subset of 22 participants also completed a task of face-based identity discrimination, the odd-identity-out task (Hodgetts et al., 2015, Lee et al., 2008). In this task participants were presented with a series of face ‘triads’ and were instructed to select the odd-one-out as quickly and as accurately as possible. On each trial, two of the faces were the same individual presented from different viewpoints, and the target (‘oddity’) was a different face presented from a different viewpoint. An equal number of targets appeared at each screen position in the triad (i.e., top centre; bottom left; bottom right). Face stimuli were greyscale photographs of adult faces, half of whom were male. Individual faces were overlaid on a black background (see Fig. 2B). Stimuli were presented using Presentation® software (Version 18.0, Neurobehavioral Systems, Inc., Berkeley, CA, www.neurobs.com). Fifty-five trials were completed, each trial (1 triad) was presented for 1500 ms, inter-stimulus interval =500ms, but no response time limit was imposed. Performance was quantified as the percentage of trials answered correctly.

##### 2.4.1.3 Experiment 2

Participants completed the tasks described here as part of a larger testing battery. In addition to the RMET and odd-identity-out tasks described above, participants in Experiment 2 additionally completed an odd-emotion-out task (Palermo et al., 2013), described below.

##### 2.4.1.4 Facial emotional expression discrimination

In the odd-emotion-out task (Palermo et al., 2013), a triad of faces was presented on every trial. Each face within each triad was of a different individual, each triad was matched on gender and had the same viewpoint (either full-face, left-facing three-quarter or right-facing three-quarter pose). Face stimuli consisted of full-colour images of individuals from the Karolinska Directed Emotional Faces database (KDEF) (Lundqvist et al., 1998). Each face was enclosed in an oval that excluded most of the hair (see Fig. 2C for an example), and distracting facial blemishes were airbrushed out. Expressions included were the 6 so-called “basic emotions” (happiness, sadness, surprise, anger, disgust and fear) (Ekman, 1992). Two faces within each triad show the same emotional expression while the third shows a different emotional expression (See Fig. 2C). Each target (‘oddity’) face was placed within a triad with two other faces displaying an emotion with which the target emotion is maximally confused (Young et al., 1997) (e.g. a disgust ‘oddity’ face paired with 2 angry foils). An equal number of target faces appeared at each screen position in the triad (i.e. left; centre; right). In order to encourage processing of facial expressions, on each trial targets and foils were matched on low level features such that, for example, open-mouthed surprise faces were paired with open-mouthed happy expressions. Participants were asked to identify the face displaying the ‘odd-one-out’ emotion expression.

Triads were presented for 1500 ms, inter-stimulus interval=500ms, and participants were instructed to respond as quickly and accurately as possible, but no limit was imposed for response time. There were 100 trials in total and an accuracy score of percentage of trials correct was calculated. Stimuli were presented using Presentation® software (Version 18.0, Neurobehavioral Systems, Inc., Berkeley, CA, www.neurobs.com). This task has previously been shown to be a reliable and sensitive measure of individual differences in face emotion processing in the typical adult population, without being susceptible to floor or ceiling effects (Palermo et al., 2013).

### 2.5 Statistical analysis

For both experiments, exploratory data analysis was carried out to assess the normality of the data distribution and to check for outliers. No variables diverged substantially from normality and therefore parametric statistical analyses were conducted. Directional Pearson's correlation coefficients were calculated to determine the relationship between mean FA values for each tract and performance on the behavioural measures of interest. As we assessed both left and right hemisphere UF, Pearson's correlations were Bonferroni-corrected by dividing α = 0.05/2 = 0.025. For the statistical test of the difference between two correlation coefficients obtained from the same sample, with the two correlations sharing one variable in common, a directional Steiger Z-test was used (Steiger, 1980), implemented within an online calculator (Lee and Preacher, 2013).

In line with the recommendations of Dienes and McLatchie (2017), complementary default JZS Bayes factors were computed for each p-value (Wetzels and Wagenmakers, 2012). The Bayes factor, expressed as BF_10_ grades the intensity of the evidence that the data provide for the alternative hypothesis (H1) versus the null (H0) on a continuous scale. A BF_10_ of 1 indicates that the observed finding is equally likely under the null and the alternative hypothesis. A BF_10_ much greater than 1 allows us to conclude that there is substantial evidence for the alternative over the null. Conversely BF_10_ values substantially less than 1 provide strong evidence in favour of the null over the alternative hypothesis (Wetzels et al., 2011). For example, a BF_10_ of 10 indicates that the finding is 10 times more likely to have occurred under the alternative hypothesis. Analogously, a BF_10_ of 0.1 is the same as a BF_01_ of 10 (i.e. 1/0.1 = 10) and indicates that the finding is 10 times more likely to have occurred under the null hypothesis (Wetzels and Wagenmakers, 2012). Some authors have suggested discrete categories of evidential strength (such that, for example a BF_10_ between 3 and 10 indicates “substantial” evidence for H1 and a BF_10_ between 1/3 and 1/10 indicates “substantial” evidence for H0), but it is important to emphasise the arbitrary nature of these labels and the continuous nature of the Bayes factor (Wetzels and Wagenmakers, 2012.)

For all tests of association, the alternative hypothesis (H1) specifies that the correlation is positive (BF_+0_). Default Bayes Factors and 95% Bayesian credibility intervals (CIs) were calculated using JASP [version 0.8] (https://jasp-stats.org).

## 3 Results

### 3.1 Experiment 1

#### 3.1.1 Behavioural performance

Participants’ total scores on the RMET (M = 81.2%, SD = 7.9%, range 61–94%) were in line with those previously reported in similar samples (Baron-Cohen et al., 2001), as were the scores on the odd-identity-out task (M = 88%, SD = 7%, range 70–96%) (Hodgetts et al., 2015). Scores on the RMET and odd-identity-out task were not significantly correlated (r=−0.007, *p* = 0.975, BF_+0_ = 0.258, 95% CI = −0.006, 0.444), indicating that performance on the two tasks is dependent on at least partly separable cognitive processes (Palermo et al., 2013).

#### 3.1.2 Uncinate Fasciculus microstructure

The UF and CST were reconstructed from both hemispheres for all participants. FA scores of the UF (M = 0.420, S.D = 0.0265, range = 0.347–0.470) were in line with previous work (Metzler-Baddeley et al., 2011). Hemispheric asymmetry (typically right > left) in the UF has been previously observed for both volume and FA (Hau et al., 2016, Thomas et al., 2015), although not all studies find such asymmetry (Thiebaut de Schotten et al., 2011). Here, a within subjects *t*-test revealed that right UF FA_T_(M = 0.425, SD = 0.025) was significantly greater than left UF FA (M = 0.415, SD = 0.031), (t(41) = 2.38, *p* = 0.022, BF_+0_ = 4.098, Hedges’ g_av_ = 0.349) (Lakens, 2013). Given this, together with the broad consensus in favour of a RH bias in emotion expression processing (Adolphs, 2002, Borod et al., 2002), all analyses were carried out for the left and right UF separately.

#### 3.1.3 Structure-behaviour associations

##### 3.1.3.1 Reading the Mind in the Eyes Test

A significant positive correlation was found between performance on the RMET (total accuracy score) and FA in the right (r = 0.421, *p =* 0.003, BF_+0_ = 15.93, 95% CI = 0.132, 0.629*)* but not the left UF (r = 0.123, *p* = 0.219, BF_+0_ = 0.399, 95% CI = −0.010, 0.413). The correlation between right UF FA and RMET was significantly stronger than that between left UF FA and RMET (z = 2.00, *p* = 0.023), consistent with RH dominance.

To determine whether the relation observed between right UF and RMET was anatomically specific, we examined the correlation between RMET performance and FA of the CST. There was no significant correlation between RMET performance and FA in either the right (r=0.080, *p*=0.308, BF_+0_=0.299, 95% CI=−0.007, 0.384) or left CST (r=0.049, *p=*0.110, BF_+0_=0.248, 95% CI=−0.006, 0.364). Importantly, the correlation between RMET performance and FA of the right UF was significantly stronger than that between RMET performance and FA in the right CST (z=2.05, *p*=0.02).

##### 3.1.3.2 Reading the Mind in the Eyes Test: emotional vs. neutral items

**Fig. 3:**
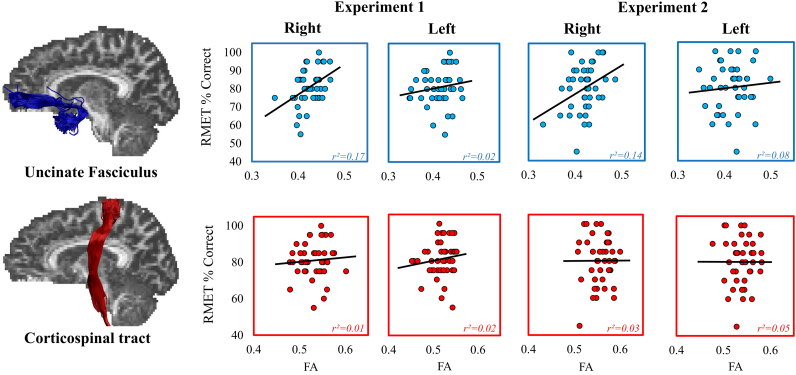
The association between fractional anisotropy (FA) in the uncinate fasciculi and corticospinal tracts and performance on the emotional trials within the Reading the Mind in the Eyes Task (RMET) for both Experiment 1 and Experiment 2. Best fitting linear regression lines are displayed on each scatter plot.

As mentioned in the methods section, RMET contains items that require decoding both affective and cognitive mental states, a division which has been employed in previous research. In particular, the amygdala appears critical for processing emotional, but not the cognitive (emotionally neutral) RMET items (Adolphs et al., 2002; Rice et al., 2014). Thus, analyses were run to further investigate whether the relation observed between FA in the right UF was unique to emotional expressions or was common to both emotional and neutral expressions. Performance for the neutral items of the RMET did not show any significant relationships to FA in any of the tracts of interest including right UF (r = 0.227, *p* = 0.074, BF_+0_ = 0.971, 95% CI=−0.022, 0.487, See Fig. 3). In contrast, performance on the emotional items of the RMET was significantly correlated with FA in the right UF (r = 0.416, *p*=0.003, BF_+0_ = 14.359, 95% CI=0.126, 0.625) but not the left UF (r = 0.153, *p* = 0.167, BF_+0_ = 0.500, 95% CI=−0.012, 0.434). While the correlation between right UF FA and RMET was greater for emotional vs. neutral items, this difference failed to reach statistical significance (z = 1.02, *p* = 0.154). As seen with total RMET performance, however, the correlation for emotional RMET items alone was stronger with right UF than with left UF (z = 1.765, *p* = 0.039), implying that microstructure of the right, but not the left UF, is preferentially linked to facial emotion expression decoding ability.

As mentioned in the introduction, it has been suggested that the RH might play a disproportionate role in processing only emotions with a negative valence (e.g. Reuter-Lorenz et al., 1983; Adolphs et al., 2001a). When looking at the positive and negative valence RMET separately, a significant association was observed between positive items and right UF FA (r = 0.422, *p* = 0.005, BF_+0_ = 16.17, 95% CI = 0.132, 0.630). A positive correlation between negative RMET items and right UF FA was also seen, but this failed to reach statistical significance (r = 0.263, p = 0.093, BF_+0_ = 1.432, 95% CI = 0.030, 0513). The correlation between right UF FA and positive valence RMET items was not however, significantly stronger than that for negatively valenced items (Z = −0.846, *p* = 0.199). The correlation between neutral items and right UF FA was not seen to be significantly different from the correlations between negative items (z = 0.186, *p =* 0.426) or positive items (z = 0.986, *p* = 0.162) and right UF FA.

##### 3.1.3.3 Facial identity discrimination

To determine whether observed associations might reflect a broader role for the UF in processing facial expression *and* facial identity, we examined the correlation between UF FA and performance on the odd-identity-out task. In contrast to the RMET, no significant correlations were observed between odd-identity-out task performance and FA in any of the tracts of interest (right UF: r = 0.118, *p* = 0.301, BF_+0_ = 0.415, 95% CI = −0.010, 0.511; left UF: r = 0.377, *p* = 0.042, BF_+0_ = 2.048, 95% CI=−0.042, 0.664; right CST: r = 0.197, *p* = 0.190, BF_+0_ = 0.607, 95% CI=−0.014, 0.557; left CST: r = 0.272, *p* = 0.110, BF_+0_ = 0.943, 95% CI = −0.022, 0.601). In this smaller subset of participants (n = 22/42), while a significant correlation was observed between FA in the right UF and total RMET performance (r = 0.478, *p* = 0.025, BF_+0_ = 5.596, 95% CI = 0.091, 0.725), the correlation between right UF FA and RMET was not significantly stronger than that between right UF FA and odd-identity-out performance (z = 1.217, p = 0.111), likely due to lack of statistical power.

### 3.2 Summary- Experiment 1

Microstructure (FA) of the right, but not left, UF was significantly associated with performance on the RMET, especially for emotional, relative to non-emotional, items, but was not significantly associated with performance on the odd-identity-out task. This suggests that the right UF may play an important role in the decoding of emotional content in facial expression, but be less critical to facial identity discrimination. To bolster these findings, in Experiment 2 we aimed firstly to replicate, in an independent sample, the association between right UF microstructure and performance on the emotional items of the RMET, and the non-association with odd-identity-out performance. In addition, one potentially confounding aspect of the previous experiment was the presence of an explicit labelling requirement in the RMET that was not present in the odd-identity-out task. To account for this procedural difference, Experiment 2 included a facial odd-emotion-out discrimination task that is more analogous to the odd-identity-out task used in Experiment 1. This allowed us to determine whether the correlation seen between facial emotion decoding and FA within the right UF still remained when there was no overt requirement for participants to name the emotion expressed in the face.

### 3.3 Experiment 2 Results

#### 3.3.1 Behavioural performance

Odd-identity-out performance for one individual was clearly an outlier, sitting almost 5 standard deviations below the mean and over 3 standard deviations below the nearest datapoint. This data point was removed prior to analysis of data for Experiment 2. As in Experiment 1, all variables of interest were normally distributed and thus parametric analyses with Pearson's r were conducted.

While performance on the RMET was slightly lower in Experiment 2 than in Experiment 1, and scores showed greater variability (M = 79.9%, S.D = 9.7, range 56–97%), performance did not significantly differ between Experiment 1 and 2 (t = 0.671(84), *p* = 0.504, BF_10_ = 0.274). Participant performance on the odd-identity-out task was also very similar across experiments (M = 89%, S.D = 6.7%, range 70–100%), and did not significantly differ (t(63) = −0.687, *p =* 0.495, BF_10_ = 0.324). Performance on the odd-emotion-out task (M = 72.9%, S.D = 6.4%) was in line with that reported in previous work (Palermo et al., 2013).

A significant positive correlation was observed between total performance on RMET and the odd-emotion-out task (r = 0.457, *p* = 0.001, BF_+0_ = 30.40, 95% CI = 0.166, 0.657), indicating that these two tasks involve highly similar cognitive processes and suggesting that performance on the RMET is strongly linked to facial emotion discrimination abilities. This correlation was additionally observed between the emotional items of the RMET and odd-emotion-out performance (r = 0.357, *p* = 0.022, BF_+0_ = 4.817, 95% CI = 0.073, 0.585).

In line with Experiment 1, no significant correlation was observed between total RMET performance and scores on the odd-identity-out task (r = 0.162, *p* = 0.150, BF_+0_ = 0.539, 95% CI=−0.013, 0.437) or between the emotional items of the RMET and odd-identity-out task (r = 0.162, *p* = 0.298, BF_+0_ = 0.543, 95% CI = −0.013, 0.438). In contrast, a significant correlation was found between performance on the odd-identity-out task and that on the odd-emotion-out task (r = 0.460, *p =* 0.001, BF_+0_ = 29.296, 95% CI = 0.166, 0.661), suggesting that these tasks require some shared perceptual mechanisms of face processing (Palermo et al., 2013). These patterns of dissociation and association between tasks are similar to those seen in a previous study (Palermo et al., 2013).

#### 3.3.2 UF microstructure

UF FA values were highly similar to those obtained in Experiment 1 (M = 0.418, S.D = 0.0315, range = 0.331–0.499). FA values were not significantly different from those seen in Experiment 1 for either right UF (t = 0.716(84), *p* = 0.476, BF_10_=0.282) or left UF (t = −0.211(83), *p* = 0.833, BF_10_ = 0.231). A within subjects *t*-test was again run comparing FA in the right versus left UF. Unlike in Experiment 1, while right UF values were numerically greater than left, no significant difference was seen between FA in the right (M=0.421, SD = 0.0 29) and left (M = 0.416, SD = 0.0344) UF in this sample (t(42) = 1.00, *p* = 0.321, BF_+0_ = 0.440, Hedges’ g_av_ = 0.157). Nevertheless, analyses were run for each hemisphere separately due to strong predictions about RH dominance for facial expression decoding.

#### 3.3.3 Structure-behaviour associations

##### 3.3.3.1 Reading the Mind in the Eyes Test (RMET)

Consistent with Experiment 1, a significant association was observed between FA in the right UF and scores on the emotional items of the RMET (r = 0.371, *p* = 0.007, BF_+0_ = 7.246, 95% CI=0.091, 0.588, see Fig. 3), further supporting the claim that emotional items within the RMET drive the relationship with right UF microstructure. Furthermore, no significant association was observed between FA in the right UF and performance on the neutral items within the RMET (r = −0.132, *p*=0.394, BF_+0_=0.108, 95% CI = −0.003, 0.254). These two correlations were seen to be significantly different from one another (z = −2.774, *p*=0.003). In-line with Experiment 1, there was no significant relationship between FA in the left UF and RMET total score (r = −0.078, *p* = 0.310, BF_+0_ = 0.134, 95% CI = −0.003, 0.286) or performance on emotional RMET items (r = 0.082, *p =* 0.600, BF_+0_ = 0.302, 95% CI = −0.007, 0.382). Indeed, a significant difference was seen in the correlation between RMET performance and FA in the right as compared to FA left UF for both RMET total score (z = 1.878, *p* = 0.030), and the emotional sub-scale score (z = 2.06, *p* = 0.020), consistent with a right lateralization of this association.

In contrast to Experiment 1, when separating emotional RMET trials by valence, a significant association was observed between negative items and right UF FA (r = 0.410, p=0.003, BF_+0_ = 14.96, 95% CI = 0.127, 0.617), but the correlation between positive items and right UF FA failed to reach statistical significance (r = 0.161, p = 0.149, BF_+0_ = 0.538, 95% CI = 0.013, 0.433). The difference between these correlations just failed to reach significance (Z = 1.463, *p* = 0.072). As in Experiment 1, no statistically significant relationship was observed between right UF FA and performance on the neutral items (r = −0.132, *p* = 0.803, BF_10_ = 0.267, 95% CI = −0.400, 0.167). The correlation between negative items and right UF FA was stronger than with neutral items (z = 2.58, *p* = 0.005) as was the correlation between positive vs. neutral items and right UF FA (z = 1.91, *p* = 0.028).

In line with the findings of Experiment 1, no significant association was observed between scores on the emotional items of the RMET and FA in either right (r = 0.053, *p* = 0.367, BF_+0_ = 0.250, 95% CI=−0.2480, 0.344) or left CST (r = 0.025 *p* = 0.435, BF_+0_ = 0.215, 95% CI=−0.274, 0.320). As in Experiment 1, the correlation between performance on the emotional items of the RMET and FA of the right UF was significantly stronger than that between Emotional RMET performance and FA of the right CST (z = 1.726, *p* = 0.04).

##### 3.3.3.2 Emotion odd-one-out discrimination

**Fig. 4:**
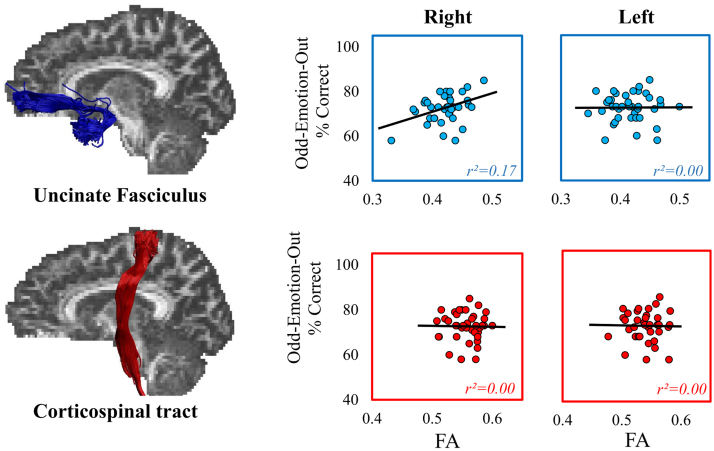
The association between Fractional Anisotropy (FA) in the uncinate fasciculi and corticospinal tracts and performance on the Odd-Emotion-Out task. Best fitting linear regression lines are displayed on each scatter plot.

Consistent with the hypothesis that the emotion decoding requirements of the RMET drive its association with right UF microstructure, a significant correlation was observed between odd-emotion-out discrimination performance and FA in the right (r = 0.413, *p* = 0.004, BF_+0_ = 6.277, 95% CI = 0.120, 0.639) but not left UF (r = 0.054, *p =* 0.371, BF_+0_ = 0.207, 95% CI = −0.262, 0.359, see Fig. 4). Further, these correlations significantly differed from one another (z = 2.407, *p* = 0.008). Supporting the anatomical specificity of these effects, there were no significant relationships between performance on the odd-emotion-out task and FA in either the left (r = −0.043, *p* = 395, BF_+0_ = 0.201, 95% CI = −0.346, 0.268) or the right CST (r = −0.055, *p* = 0.367, BF_+0_ = 0.206, 95% CI = −0.356, 0.257). Indeed, a comparison of the correlations between FA in the right UF and CST showed that the correlation between emotion oddity performance and FA in the right UF was significantly stronger than the correlation with the right CST (z = 2.472, *p* = 0.007).

##### 3.3.3.3 Face identity discrimination

As in Experiment 1, there was no significant correlation between performance on the odd-identity-out task and FA in either right (r = 0.079, p = 0.307, BF_+0_ = 0.215, 95% CI = −0.227, 0.371) or left UF (r = −0.181, p = 0.126, BF_+0_ = 0.362, 95% CI = −0.459, 0.130). To determine whether this absence of significant relationship between right UF and odd-identity-out performance was indeed meaningfully different from the significant relationships seen with the emotion-based tasks, comparisons were run between this correlation and the correlations previously reported between right UF and each of the other cognitive tasks. The relationship between odd-identity-out task and FA in the right UF was indeed seen to be significantly different from the correlation between right UF FA and performance on the odd-emotion-out task (z = 2.193, *p* = 0.014), and approached significance for the correlation with emotion items in the RMET (z = 1.522, *p* = 0.064). Thus, findings from this larger sample support the hypothesis that the association seen with the right UF was specific to facial *emotion* processing.

##### 3.3.3.4 Bayes Multiple Regression

Finally, to examine whether performance on emotion labelling in the RMET and emotion discrimination performance from the odd-expression-out task were independently related to microstructure of the right UF, we performed Bayesian multiple regression and model comparison using JASP (Rouder and Morey, 2012).

Bayesian regression indicated that the best fitting model was one that took into account both RMET emotional labelling *and* odd-expression-out performance as predictors (BF vs. null model = 8.367). The next best model contained odd-emotion-out discrimination only (BF vs. null model = 6.309). A model just containing RMET emotion labelling performance was also supported (BF vs. null model = 4.532). These findings provide strong evidence that the relation between right UF microstructure and emotion decoding is not restricted to tasks requiring overt emotion labelling as in the RMET.

## 4 Discussion

A critical question for affective neuroscience is how variability in the structural organization of the human brain impacts facial emotion decoding - a critical skill linked to real world social competency, empathic tendencies and the success of interpersonal relationships (Elfenbein et al., 2007, Lewis et al., 2016). Across two experiments, we established the presence of a robust correlation between facial emotion processing abilities and the microstructural organization of the right Uncinate Fasciculus (UF), but not of the left UF, or of a control tract implicated in complex motor skills (the CST) (Engel et al., 2014). In Experiment 1, we found a positive correlation between right UF FA and performance on the well-known ‘Reading the Mind in the Eyes Test’ (RMET), which was driven predominantly by the emotional items within the task. Experiment 2 corroborated and expanded this finding, replicating the positive correlation between right UF microstructure and performance on the emotional items of the RMET, and additionally revealing a positive correlation between right UF FA and odd-expression-out performance. Further, across both experiments, we found little evidence of a relation between UF microstructure and performance on an odd-identity-out face discrimination task. Our findings thus indicate that the observed relations between right UF microstructure and emotion-based tasks reflect the emotion processing components of the tasks and not more general face-processing abilities. Taken together, our findings highlight the important role of right-hemisphere frontotemporal connectivity, supported by the UF, in underpinning facial emotion processing.

In humans and non-human primates, UF creates a direct structural connection between portions of the anterior and medial temporal lobes (ATL) and sectors of the orbital and medial prefrontal cortices (OMPFC) (Schmahmann et al., 2007, Thiebaut de Schotten et al., 2012). In humans, lesion studies have found that damage to both the ATL (including the amygdala, rhinal cortex and temporal pole) and OMPFC, especially in the right hemisphere (RH), can result in impaired facial expression processing on a variety of tasks (see introduction for references). Findings from lesion studies however, have been variable (see Adolphs, 2002; Willis et al., 2014, for discussion). Interpretation of these lesion studies is additionally complicated by (a) the fact that different face stimuli, tasks (variously intensity rating, forced-choice labelling, matching or categorization tasks), and analysis strategies have been used across studies; and (b) the inclusion of patients with differing lesion aetiologies (variously including epilepsy, herpes simplex encephalitis, stroke and traumatic brain injury). Our findings thus add significantly to the existing literature on the neuroanatomy of facial expression processing.

It has been suggested that some neural regions might be disproportionately involved in the processing of specific facial expressions. While some previous studies emphasise a role for the amygdala in processing facial expressions of fear specifically (Adolphs et al., 1995, Calder et al., 2001), patients with bilateral amygdala damage typically show facial emotion processing impairments across several negative emotions (e.g. Adolphs et al., 1999). Studies of patients with static OMPFC damage (Hornak et al., 1996), specifically orbital and ventromedial regions (Heberlein et al., 2008, Tsuchida and Fellows, 2012), and of patients with static ATL damage extending beyond the amygdala (Adolphs et al., 2003, Adolphs et al., 2001b, Anderson et al., 2000, Schmolck and Squire, 2001) reveal a broader pattern of deficits in the recognition of several, especially negative, basic emotions. Patients with progressive ATL and OMPFC degeneration resulting from FTD, however, can additionally show impairments even in the recognition of happy faces (Rosen et al., 2002). Far less work has investigated the neural underpinnings of complex social emotion recognition, although one study found impaired recognition (labelling) of social emotions such as embarrassment in patients with orbitofrontal cortex damage resulting from traumatic brain injury (Beer et al., 2003).

A wide variety of tasks and stimuli have been used in previous studies of emotion processing, and so here, studies of patients with frontal and temporal lobe lesions on analogues of the odd-expression-out and RMET are particularly pertinent. One study of RMET in patients with (bilateral) amygdala damage (Adolphs et al., 2002) found impairments only on the emotional and not the cognitive items of the RMET. This finding was not replicated by Shaw et al. (2005), however, who found that damage to either left or right amygdala was associated with impaired recognition of both emotional and cognitive items. This latter study did find, however, that right (vs. left) OMPFC lesions were associated specifically with impairment on negative valence RMET items. Stone et al. (2003) found that amygdala damage had a greater impact on emotional versus cognitive RMET items and suggested that the non-emotional items on the RMET (e.g. ‘insisting’) can be solved by judging gaze direction alone, without requiring fine grained judgments of facial expression. Relatedly, the RMET has been suggested to be best viewed as a sensitive indicator of individual differences in facial emotion recognition, rather than of more abstract ‘mindreading’ processes (Oakley et al., 2016). Our findings point to an important role for frontotemporal connectivity, particularly in the RH, in processing a range of basic and complex emotions. They are also consistent with suggestions that RH frontotemporal connectivity is particular important for the decoding of emotional relative to cognitive mental states from faces (Sabbagh, 2004, Shamay-Tsoory et al., 2009).

To our knowledge, the odd-expression-out discrimination task used here has not been the subject of neuropsychological investigations. Willis et al. (2014), however, previously found that facial emotion expression matching, particularly for subtle (morphed) expressions of basic emotions, was impaired following orbitofrontal cortex damage. Similarly, in one case of bilateral damage to the amygdala, impairment was seen not only on the RMET (Stone et al., 2003), but also on a test of facial expression matching similar to the odd-expression-out task (Young et al., 1995). In addition, patients with FTD show impairments both on the RMET (Gregory et al., 2002) and tasks of facial emotion matching (Rosen et al., 2002, Rosen et al., 2006), both of which have been linked to the extent of RH temporal and orbitofrontal cortical atrophy (Rosen et al., 2006). These results likewise support our finding of a strong correlation between performance on the RMET and the emotion oddity task, both of which were associated with right UF microstructure, and suggest that RH frontotemporal connectivity is important both for facial emotion discrimination and labelling skills.

Converging evidence for a role for RH ATL and OMPFC in both facial emotion labelling and perceptual discrimination tasks comes from functional imaging, including studies of analogues of the RMET (Calder et al., 2002, Adams et al., 2010, Wicker et al., 2003) and of simultaneous matching to sample tasks using expressive faces (LoPresti et al., 2008). Furthermore, right ATL (rhinal cortex) shows *perceptual* adaptation effects for facial expressions (Furl et al., 2007). When directly contrasted, explicit facial emotion labelling tasks typically result in increased OMPFC activity (particular in ventrolateral PFC), relative to tasks with no overt labelling requirement (see Dricu and Fruhholz, 2016 for meta-analysis). Our findings extend this work to indicate that right-hemisphere frontotemporal intercommunication, enabled by the UF, is critical for perceptual and conceptual facial emotion processing, consistent with distributed network models (e.g. Adolphs, 2002; Vuilleumier and Pourtois, 2007).

Whilst our findings provide strong evidence that right UF microstructure was related to facial emotion processing, we found little evidence that UF microstructure was important for facial identity processing, as no significant correlation was observed between right UF FA and odd-identity-out performance. This absence of a relationship between UF microstructure and facial identity matching is consistent with a recent finding (Alm et al., 2016). In addition, facial identity matching performance can be spared in patients with frontal and temporal lobe lesions who exhibit deficits in facial emotion processing (Hutchings et al., 2017), although identity and expression tasks are often not comparably difficult in such studies, complicating findings (Calder and Young, 2005). Nevertheless, while neither right UF microstructure nor RMET performance was related to odd-identity-out performance, odd-identity-out and odd-expression-out performance was correlated across individuals (see also Braun et al., 1994; Palermo et al., 2013). In addition, evidence in favour of the null (i.e. no correlation between UF microstructure and face identity discrimination), as indicated by Bayes factors, was only substantial in Experiment 2. Our findings are thus perhaps most consistent with accounts positing a partial or graded, rather than absolute, segregation of facial identity and expression processing in the brain (Calder and Young, 2005, Dahl et al., 2016), with processing in more anterior brain regions weighted towards expression processing.

The suggestion of graded specialization for expression versus identity processing in frontotemporal regions dovetails with work on the coding properties of more anterior sectors of the monkey ‘face patch’ network - a set of interconnected regions that show stronger fMRI activation to faces than to other classes of object. Tsao et al. (2008) identified discrete regions of face-selective cortex in OMPFC that responded more strongly to expressive than to neutral faces. They also found that face patches in ATL were modulated by facial expression, though less so than frontal face patches. Notably, one of the frontal face patches was strongly lateralized to the RH, consistent with the RH bias for face expression processing seen in nonhuman primates (Lindell, 2013). Other neuronal recording studies of monkey OMPFC and ATL (including amygdala) report activity related to facial expression, but also in some instances identity processing and the joint coding of expression and identity (see Rolls, 2015; Rutishauser et al., 2015; for reviews). The precise nature of the anatomical connectivity between frontal and temporal face patches in the monkey brain is unclear (Grimaldi et al., 2016), but the UF, whose anatomy is highly conserved between humans and monkeys (Thiebaut de Schotten et al., 2012), is well placed to mediate such connections, either directly or indirectly.

The finding that (right) UF microstructure was *independently* related to both RMET and odd-expression-out performance is consistent with the notion of a distributed, multicomponent RH cortically based affect-processing network (Bowers et al., 1993), underpinned by the UF. The question arises as to the precise functional contribution of frontotemporal connectivity to expression decoding skills. The UF may participate in somewhat different functions, including (1) enabling fine-grained visual representations of the perceptual properties of facial expressions (important for odd-expression-out discrimination of highly similar expressions with overlapping features, such as anger and fear) and (2) retrieving conceptual knowledge about the emotion that those stimuli signal, plus lexical knowledge necessary for linking an expression to a verbal label (as required in the RMET) (Adolphs, 2002). Perceptual and conceptual processes may also interact, as emotion concept knowledge may help support the normal perception of discrete emotion categories (Lindquist et al., 2014). Such a role for the right UF is consistent with the notion of an extended ventral visual processing stream running into ventrolateral PFC (Kravitz et al., 2013), in which cascaded, interactive feedforward and feedback processing may mediate the challenging visual perceptual discrimination of facial expression (Murray et al., 2017). A role for the UF in the storage and retrieval of emotion concepts is also consistent with an account of the neural substrates of semantic cognition based on the idea of a category-general semantic ‘hub’ located in ATL (Lambon-Ralph et al., 2017). On this account, the ATL, by virtue of its unique connectivity, functions to bind information from modality-specific systems to form and store coherent, transmodal, generalizable concepts, including social and emotional concepts (Lambon-Ralph et al., 2017). A number of studies from Lambon Ralph and colleagues have indeed shown that ATL is critically important for the processing of social concepts (e.g. Binney et al., 2016; Pobric et al., 2016). Consistent with our findings, however, previous work has additionally highlighted potentially distinct sensory versus semantic processing regions in ATL (Olson et al., 2013).

There remains some conflict within the literature on the hemispheric specialization of facial emotion decoding, although the majority view is that RH plays a dominant role (Borod, 2002; Damasio and Adolphs, 2003; Gainotti, 2012). This “right hemisphere hypothesis” is supported by the results of (a) behavioural studies on healthy participants using the divided visual field and chimeric faces techniques (Lindell, 2013, Najt et al., 2012); (b) studies in split-brain patients (Benowitz et al., 1983); and (c) large-scale and meta-analytic studies of the effects of focal (Adolphs et al., 1996, Adolphs, 2000; Abbott et al., 2013) and neurodegenerative (Binney et al., 2016) right vs. left hemisphere brain damage. Nevertheless, functional neuroimaging suggests at least some involvement of left hemisphere structures in face emotion processing (Murphy et al., 2003, Dricu and Fruhholz, 2016), and studies that compared patients with lesions in either the LH or RH sometimes indicate that both hemispheres are involved in recognizing emotional expressions, albeit not to the same extent (e.g. Abbott et al., 2013). These findings indicate that RH specialization for emotion expression decoding is graded, rather than absolute (see also Behrmann and Plaut, 2013).

Despite this broad consensus, the nature and developmental origins of the RH dominance in facial emotion processing, and indeed facial processing in general, are still largely unclear, with several factors likely to contribute (Adolphs, 2002, Behrmann and Plaut, 2013). Neuroanatomically, our findings indicate that hemispheric asymmetry in UF microstructure is one such contributing factor. Previous work on the role of right ATL in semantic cognition has suggested that hemispheric differences in patterns of anatomical connectivity of UF are also important. For example, according to the ATL semantic hub model (Lambon Ralph et al., 2017), social and emotional concepts, like other concepts, are supported bilaterally across the ATL, but regions within the right temporal pole (TP) contribute more to social and emotional concepts than their LH counterparts. This occurs by virtue of increased RH TP connectivity to networks that support social perception and valence coding, relative to LH TP (which is more strongly connected to left hemisphere language centres). Recent dMRI tractography studies indeed suggest greater RH connectivity (indexed by number of streamlines) between OMPFC and TP cortex, mediated by UF fibres (Hau et al., 2016, Papinutto et al., 2016).

One much debated issue concerns the extent to which RH might play a disproportionate role in processing all emotions or only emotions with a negative valence; with processing of positive emotion relying on LH (Reuter-Lorenz et al., 1983), or bilaterally (Adolph et al., 2001a). The uncertainty regarding valence may be due to methodological differences, including participants’ lesion type, severity, and chronicity in neuropsychological studies, as well as variations in the tasks and stimuli used to assess facial emotion decoding. Typically, studies are imbalanced in terms of the number of positive and negative expressions tested (i.e., one positive – happy - versus several negative). Our findings with the RMET, which contains several different positive and negative expressions, are not consistent with a valence hypothesis, but clearly indicate that the RH is disproportionately important in the processing of face emotion. It remains a fundamental question why emotion processing should feature hemispheric lateralization.

From the current findings, it is unclear whether the observed effects are specific to facial emotion or whether they may hold for other modalities of emotion expression. OMPFC and ATL damage has been seen to result in impairments in the perception of emotion from modalities other than faces, including from vocal and bodily expressions (e.g. Adolphs et al., 2001b; Hornak et al., 2003; Keane et al., 2002), suggesting that the UF may be involved in transmodal aspects of emotion processing. Future studies should consider investigating the generalisability of the current findings to additional emotion expression modalities to verify this presumption. This point also highlights the important issue of the extent to which the right frontotemporal network underpinned by the UF might be specialized for processing salient or behaviourally significant stimuli, rather than emotion expressions per se (Ranganath and Ritchey, 2012). The function of this network might best be construed in more basic terms, for example, linking perceptual representations of stimuli with their punishing or rewarding contingencies (Rolls, 2015, Adolphs, 2014, Von Der Heide et al., 2013). With regard to emotional behaviour, decoding and rapidly readjusting the reinforcement value of visual signals is likely to be crucial: highly similar smiles may variously communicate relaxation, concern, or even contempt, each with very different rewarding or punishing contingencies (Rychlowska et al., 2017). Indeed other evidence suggests that RH OMPFC is critically involved in deciphering socioemotional information to enable socially appropriate behaviour (Goodkind et al., 2012, Kringelbach and Rolls, 2003). A similar role may hold for regions of ATL, including amygdala (Murray et al., 2017) but also rhinal cortex (Meunier et al., 2006). A related issue for future investigation is whether the UF is differentially involved in the processing of static, relative to dynamic, facial emotional expressions. There is some evidence that recognition of dynamic expressions can be spared following damage to right frontotemporal cortex (Adolphs et al., 2003), with those authors suggesting that dorsal stream structures might play an important role in the decoding of emotions expressed via facial motion (see also Freiwald et al., 2016).

The causes of inter-individual variation in white matter microstructure are not fully understood, but likely include a complex interplay between genetic and environmental factors over the lifecourse. The microstructure of the UF has been shown to be highly heritable (Budisavljevic et al., 2016), and in a recent study, microstructure of the right UF measured at just 6 months of age predicted infants’ reciprocal joint attention skills at 9 months of age (Elison et al., 2013). At the same time, the UF is a relatively late maturing tract, showing microstructural alteration into the fourth decade of life (Lebel et al., 2012) (notably in synchrony with the late maturation of facial emotion recognition abilities, Hartshorne and Germine, 2015), suggesting that its development can also be shaped by experience. Thus, UF microstructure is likely to both shape, and be shaped by, social interaction in a transactional fashion (Gottlieb, 1991).

According to Chakrabarti and Baron-Cohen (2006), a very early developing, and presumably partly innately specified (Ekman, 1992) neural “emotion detector” is critical for the development of social attention mechanisms, including reciprocal joint attention, which in turn over development feed into the ability to empathize with other individuals. Notably, a recent structural equation modelling study of healthy adults (Lewis et al., 2016), found that the ability to recognize visually-presented emotion reflects both distinct and overlapping processes, operating at different levels of abstraction, including emotion (e.g. fear)-specific factors, but also emotion-general face-specific factors. The face-specific component of emotion recognition (linked by our findings to right UF microstructure) was associated with self-report empathy, which is striking, given that lesions to the right UF lead to a reduction in self-report empathy (Oishi et al., 2015, Herbet et al., 2015). Collectively, these findings provide an account as to why both early and late developing traits and disorders including ASD (Ameis and Catani, 2015); Psychopathy (Sobhani et al., 2015) and FTD (Mahoney et al., 2014), which feature disrupted right UF microstructure, are all associated with alterations in both emotion recognition *and* empathy.

The biological interpretation of interindividual differences in FA is challenging, since FA may vary due to a plethora of functionally relevant biological properties of white matter such as myelination; membrane permeability; and axonal number, diameter and configuration (Jones et al., 2013), each of which may differently impact on the transmission of information between neural regions and be influenced by distinct genetic and environmental factors. One recent study found strong correspondence between myelin microstructure and DTI microstructural indices, where high FA was linked to high myelin density and a sharply tuned histological orientation profile (Seehaus et al., 2015). Such underlying microstructural properties are important for facilitating information transmission between distributed neural regions. For instance, activity-dependent variation in axon myelination may support synchronised functional coupling between distal brain regions by regulating conduction velocities (Fields, 2008, Bells et al., 2017). Future work should use emerging methods to estimate microstructural properties, such as axonal density and myelin water fraction (Lerch et al., 2017) in the UF and determine their relationship to emotion expressing processing.

In summary, we found, across two studies, that individual differences in the microstructure of the right UF, a structure whose function has remained rather enigmatic (Von Der Heide et al., 2013), predicted individual differences in two distinct tasks of facial emotion processing, but was not related to individual differences in facial identity processing. This result is consistent with a role for an extended right frontotemporal network, interconnected via the UF, in the decoding of one important class of social stimuli, and may reflect a broader role in the encoding and reconstruction of the emotional value, salience and meaning of stimuli, crucially important for successfully navigating the social world.
